# An Energy-Efficient Routing Algorithm Based on Greedy Strategy for Energy Harvesting Wireless Sensor Networks

**DOI:** 10.3390/s22041645

**Published:** 2022-02-19

**Authors:** Sheng Hao, Yong Hong, Yu He

**Affiliations:** 1 School of Computer Science, Central China Normal University, Wuhan 430079, China; mikeshao@ccnu.edu.cn; 2 National Language Resources Monitoring and Research Center for Network Media, Central China Normal University, Wuhan 430079, China; 3 College of Information Engineering, Huanghuai University, Zhumadian 463000, China

**Keywords:** energy harvesting, wireless sensor network, greedy strategy, energy efficiency, reception state adjustment mechanism, routing protocol

## Abstract

Energy harvesting wireless sensor network (EH-WSN) is considered to be one of the key enabling technologies for the internet of things (IoT) construction. Although the introduced EH technology can alleviate the energy limitation problem that occurs in the traditional wireless sensor network (WSN), most of the current studies on EH-WSN fail to adequately consider the relationship between energy state and data buffer constraint, and thereby they do not address well the issues of energy efficiency and long end-to-end delay. In view of the above problems, a brand new greedy strategy-based energy-efficient routing protocol is proposed in this paper. Firstly, in the system modeling process, we construct an energy evaluation model, which comprehensively considers the energy harvesting, energy consumption and energy classification factors, to identify the energy state of node. Then, we establish a channel feature-based communication range judgment model to determine the transmission area of nodes. Combining these two models, a reception state adjustment mechanism is designed. It takes the buffer occupancy and the MAC layer protocol into account to adjust the data reception state of nodes. On this basis, we propose a greedy strategy-based routing algorithm. In addition, we also analyze the correctness and computational complexity of the proposed algorithm. Finally, we conduct extensive simulation experiments to show that our algorithm achieves optimum performance in energy consumption, packet delivery ratio, average hop count and end-to-end delay and acceptable performance in energy variance.

## 1. Introduction

Wireless sensor network (WSN) plays a significant role in constructing IoT systems, which is composed of many sensor nodes in a multi-hop self-organizing manner. Due to the properties of easy connectivity and high data rate, WSN has been widely used in diverse fields including the military, industry, agriculture and so on [1,2,3]. However, as it is battery powered, the lifetime of a sensor node is limited by the battery capacity.

With the development of green energy, energy harvesting (EH) techniques are introduced into the WSNs. Driven by the EH techniques, nodes can intake energy from the ambient environment (e.g., solar, wind, RF radiation, etc.) and recharge their batteries during operation, which can improve the energy efficiency and extend the lifetime of the network [4,5]. At present, many routing algorithms have been proposed for energy harvesting wireless sensor networks (EH-WSNs) which focus on exploiting the harvested energy and optimizing energy efficiency [6,7,8,9].

### 1.1. Motivation

Although many meaningful works have paid attention to the EH-WSNs routing protocol design [10,11,12,13,14,15,16], the energy efficiency problem is not resolved well. In detail, most of the existing studies do not fully consider the energy harvesting rate and the impact of channel features on energy consumption, as well as the energy wastage caused by buffer constraint. It should be also noted that if the status of the data buffer fails to be considered in the protocol design, the congestion, transmission time prolonging and energy-wasting issues would easier to be caused [17]. When the node has insufficient residual energy (enters the sleep mode) and the buffer is not empty (there are still packets in the buffer), it inevitably causes the transmission time of this part of packets to be prolonged. As a result, it is necessary to design a brand new routing protocol that comprehensively takes the energy state and buffer occupancy into account.

On the engineering application aspect, EH technique has been used to ease the issues of energy constraint and improve the energy balance in traditional WSN. Currently, EH-WSN has achieved great commercial success in many fields. Numerous EH-WSN based IoT devices are widely used in our lives, such as Smart Home, 5G/B5G and 6G communication, Wise information technology of med (WITMED), intelligent wearable devices, etc., which contribute to the development of society and lead our daily affairs to be convenient and intelligent. In addition, since the nodes of EH-WSN can extract energy from the surroundings, environmental pollution caused by battery replacement and network maintenance costs are correspondingly reduced. This is in line with the internationally promoted rules of 3R (reducing, reusing and recycling). Especially in our country, China, the widespread use of this technology is meaningful for achieving the goal of Carbon Neutrality [18] in the future. Hence, a thorough study of the EH-WSN routing algorithm design has practical value for constructing IoT systems.

### 1.2. Contributions

The core contributions of this paper can be summarized as follows:

- We construct an energy evaluation model, which considers the effect of energy harvesting, energy consumption and energy classification, to determine the energy state of the node. Furthermore, we also formulate a channel feature (Rayleigh fading) based communication range judgment model. Through this model, the neighbor nodes set, forward transmission nodes set and candidate nodes set of the current node can be identified.
- Gathering the above two models, we design a reception state adjustment mechanism. Through considering the buffer occupancy and IEEE 802.11b protocol hidden terminal problem, this mechanism can adjust the data reception state of nodes dynamically.
- On this basis, we propose a greedy strategy-based relay node selection algorithm, which adopts the greedy strategy to select the best next hop. At the same time, we also give a solution for the extreme case.
- We analyze the correctness and computational complexity of our algorithm.
- We conduct extensive simulation experiments to validate the superior performance of our proposed algorithm.

### 1.3. Paper Organization

The remainder of this paper is organized as follows. The related works about the WSN and EH-WSN routing protocols are discussed in Section 2. The overview of the system model, including the energy evaluation model, communication range judgment model and reception state adjustment mechanism, are presented in Section 3. The details of the proposed algorithm are introduced in Section 4. The results of the simulation experiment are shown in Section 5. Finally, we conclude this paper in Section 6.

## 2. Related Work

### 2.1. WSN Routing Algorithms

Typical routing algorithms in WSNs can be a guide for designing EH-WSNs routing protocols, as EH-WSNs are WSNs-based [11,12]. So far, numerous routing protocols for WSNs have been introduced. Greedy Perimeter Stateless Routing (GPSR) algorithm [19] is a typical geographic-aware routing protocol. It includes two types of data packet forwarding strategies, namely greedy forwarding strategy and perimeter forwarding strategy. By taking a greedy forwarding strategy, nodes select the next hop closest to the destination. At same time, in areas where greedy forwarding fails (i.e., routing hole region), the GPSR algorithm adopts the perimeter forwarding strategy (a void recovery method) to forward the packets bypassing this area, thus ensuring the stable transmission of packets. Through a void recovery method, it solves the routing hole problem where greedy forwarding fails. Low Energy Adaptive Clustering Hierarchy (LEACH) algorithm [20] is an adaptive clustering topology algorithm. It consists of two phases. In the set-up phase, the cluster head node is randomly selected using a probability function, and other nodes join the corresponding cluster following the proximity principle. In the data transmission phase, non-cluster head nodes send data to the cluster head, and then the cluster head fuses the collected data and forwards it to the sink. Ant Colony Optimization in combination with Hop Count Minimization (ACOHCM) algorithm [21] comprehensively considers the energy constraint, network load balancing and dynamic network topology, combines the ant colony algorithm and the minimum hop algorithm and selects the next hop according to the residual energy and distance. This algorithm can find the optimal routing path with low energy consumption and balanced energy consumption on each node. Optimizing routing based on a congestion control (CCOR) algorithm [17] is a congestion control algorithm. In this method, a queuing network model is established to detect the congestion degree of nodes. Moreover, it constructs a hybrid formula that considers the position, average packet service rate and congestion degree to determine the optimal next hop.

Reviewing the above algorithms, it is easy to find that WSN algorithms mainly concentrate on how to minimize energy consumption and increase the lifetime of a network. However, as inspiring as these works for traditional WSN routing protocols can be for our research, the EH-WSN routing algorithms design is still very different from these works.

### 2.2. EH-WSN Routing Algorithms

Routing algorithms in EH-WSNs mainly focus on optimizing network performance within the constraint of energy harvesting rate. In recent years, many routing protocols for EH-WSNs were also proposed. Typically, the authors of [7] proposed the Energy Harvesting Aware Ad hoc On-Demand Distance Vector Routing (AODV-EHA) protocol, which integrates the AODV protocol with energy harvesting. In this method, the “hop count” is replaced by the “energy count” (the predicted transmission cost of a packet) to find a path with the lowest transmission cost. The authors of [1] give an Effective Energy-Harvesting-Aware Routing Algorithm (EHARA). This algorithm extends the backoff period in the MAC layer IEEE 802.15.4 CSMA/CA by adding an “extra backoff” time, which can ensure the nodes have more time to collect energy and maintain the energy balance. The authors of [8] propose a Stability-Aware Geographical Routing Protocol (SAGREH). In this method, the candidate paths are determined through considering the residual energy and energy harvesting rate. Then, it utilizes the active link quality measurement to evaluate the quality of each path and chooses the transmission route with the highest reliability. The authors of [6] propose an Energy Harvesting Routing (EHR) algorithm. In this method, it constructs an energy rank model, which ranks the residual energy of nodes and selects the node with the highest energy level as candidate nodes. At the same time, it designs a node information update mechanism, which can save the extra energy consumed by the information exchange of nodes. Finally, the relay node is selected according to the energy level and energy harvesting density. The authors of [11] propose a Physarum-inspired routing protocol for EH-WSN (EHPRP). This algorithm establishes the Poiseuille equation by considering the residual energy, angle and distance factors of the node. Based on this equation, the best next hop node can be selected, which can reduce energy consumption and preserve the energy-neutral state of the network. The authors of [14] propose a routing algorithm based on the LA theory (DEH-LA-SERA). It constructs a multi-factor measurement model for sensor nodes, including node stability model, energy ratio function, expected harvesting energy model and direction judgment model, thereby deriving node weighted value to determine the next hop node. This algorithm guarantees an overall energy balance and savings while maintaining a stable selected path.

Although the EH-WSNs algorithms we mentioned above can reduce energy consumption and extend network lifetime, these algorithms overlook the problems of limited data buffer capacity and long transmission delay.

## 3. System Model

In this section, we firstly provide an energy evaluation model. Then, we construct a communication range judgement model. Finally, we design a reception state adjustment mechanism. The related symbols in this paper are listed in Table 1.

**Figure 1:**
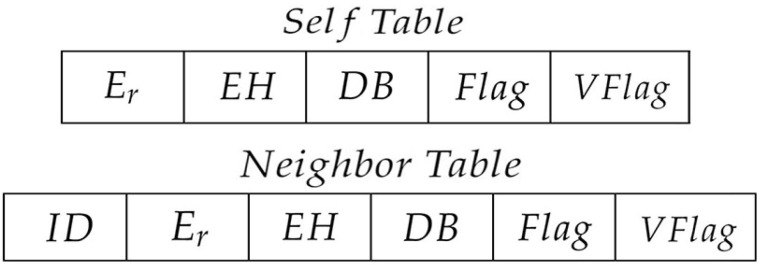
Data conservation structure.

Before the modeling process, the following assumptions should be followed:

1. *n* sensor nodes are randomly distributed in a M×M two-dimensional plane, and the positions of all nodes are settled.
2. Nodes in the network are homogeneous, where the maximum battery capacity is $E_{m}$, and all nodes have the same transmitting power.
3. Positions of all nodes (denoted by $\left(x_{.},y_{.}\right)$) can be known by position discovery techniques [22,23]. If node *j* is within the communication range of node *i* (i.e., neighbor node), a wireless link exists between these two nodes. The extreme case (i.e., the routing loop problem) would be handled in the process of routing protocol design.
4. Each node is equipped with a solar device that can harvest additional energy from the surrounding environment. The energy harvesting rate EH(t) follows a Gaussian random distribution $N(b,c^{2})$, where the detailed expression is written as $EH\left(t\right)=ae^{-\frac{{\left(t-b\right)}^{2}}{2c^{2}}}$ (*t* denotes time, and *a*, *b*, *c* represent the coefficients affected by light intensity).
5. The data buffer of each node is limited. Each node can sense its residual energy and data buffer occupancy.
6. Nodes periodically exchange state information with their neighbor nodes to update the information in its Self Table and Neighbor Table (Figure 1).

Each node in the network has five main attributes: residual energy (Er), energy harvesting rate (EH), data buffer occupancy (DB), the mark of whether the buffer can receive data (Flag) and the mark of whether the node is void node (VFlag). In addition, each node maintains two tables containing the above attributes: Self Table and Neighbor Table.

**Figure 2:**
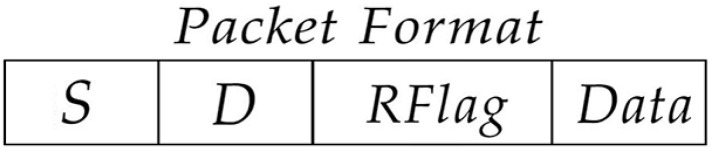
Packet format.

The format of packets generated by nodes is illustrated in Figure 2, where *S* denotes the source node address, *D* represents the destination node address and RFlag indicates the forward strategy of the packet.

### 3.1. Energy Evaluation Model

#### 3.1.1. Energy Harvesting Factor

**Figure 3:**
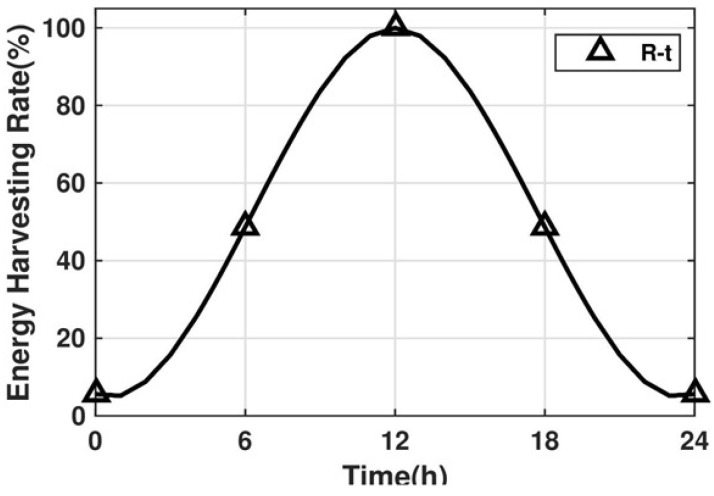
Energy harvesting rate in different time instant [6].

Energy prediction is an important issue as environmental resources are uncontrollable. Obviously, the energy harvesting rate (EH) of each node is different, which depends on the location of the node and the surrounding environment, e.g., EH of a node in the shadow must be lower than that of a node directly exposed to the sun. At the same time, EH also varies over time. Generally, EH of a node reaches the maximum at noon and the lowest at night, which is almost zero, as shown in Figure 3. We make use of Gaussian function to simulate the change curve of EH with time [6,24]:

(1) $$EH_{i}\left(t\right)=a_{i}e^{-\frac{{\left(t-b_{i}\right)}^{2}}{2c_{i}^{2}}}$$

where $a_{i},b_{i},c_{i}$ represent the coefficients affected by light intensity.

#### 3.1.2. Energy Consumption Factor

Here, we consider the energy model of the node *i* as follows:

(2) $$E_{r}^{i}=\min\left\{E_{0}^{i}+E_{h}^{i}-I\left(e\left(i\right)\right)\left(E_{tx}^{i}+E_{rx}^{i}\right)-E_{s}^{i},E_{m}\right\}$$

where I(·) is a binary marking function and $e\left(i\right)$ denotes the event that node *i* receives or sends data. If node *i* receives or sends data, I(e(i)) returns 1, otherwise, returns 0. Let the transmission power and reception power of nodes be $P_{t}$ and $P_{r}$, respectively. Then, the energy consumed by sending and receiving a packet can be expressed as:

(3) $$E_{tx}^{i}+E_{rx}^{i}=\left(P_{t}+P_{r}\right)\frac{L}{R}$$

Since the channel conditions are constantly changing, the total signal gain follows the Rayleigh distribution after reflective, refractive and scattering in multiple paths [25,26]. Manhattan’s experiment proves that Rayleigh fading channel is more suitable for signal attenuation calculation models in a realistic environment with interference factors [27]. Therefore, the received power can be further expressed as follows [28]:

(4) $$P_{r}=\ell P_{t}C{d_{ij}}^{-2}$$

(5) $$C=\frac{G_{t}G_{r}\lambda^{2}}{{\left(4\pi f_{c}\right)}^{2}}$$

where $G_{t}$, $G_{r}$ represents the transmitting and receiving antenna gain, λ denotes a wavelength, $f_{c}$ indicates the carrier frequency, *ℓ* represents Rayleigh gain factor following index distribution, in which probability density function is $f\left(\ell \right)=e^{-\ell }\left(0\leq \ell \leq \infty \right)$.

#### 3.1.3. Energy Level System

**Figure 4:**
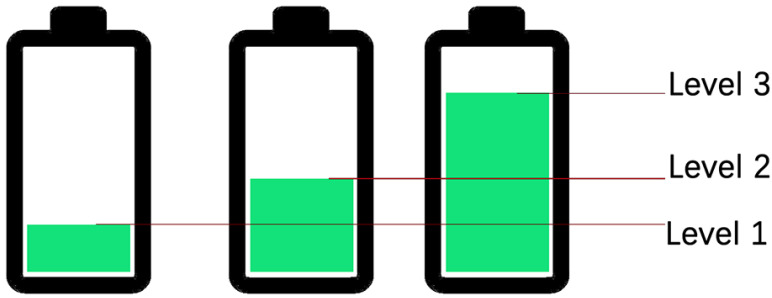
Energy level system.

To describe the residual energy of nodes more clearly, we divide the battery into three levels, as shown in Figure 4. Depending on the residual energy $E_{r}$, we study the following two cases:

1. Case1:0≤Er≤Level1
  In this case, since the node has insufficient energy to maintain more data transmission, the data receiving device would be shut down temporarily. Then, the node confirms whether there are still packets to be sent in its data buffer. If so, continue processing these remaining packets until the residual energy or number of packets reaches 0; otherwise, turn off the sending device and enter sleep mode.
2. Case2:Er≥Level2
  When the residual energy is greater than Level2, the node would be transitioned to active mode if it is in sleep mode.

### 3.2. Communication Range Judgment Model

#### 3.2.1. Neighbor Nodes

In order to identify the neighbor nodes of each node, it needs to determine the maximum communication range. The distance from node *i* to *j* can be calculated as:

(6) $$d_{ij}=\sqrt{{\left(x_{j}-x_{i}\right)}^{2}+{\left(y_{j}-y_{i}\right)}^{2}}$$

Due to the influence of multipath effect (reflection, refractive, obstacle and the influence between different signals), the transmission process leads to signal attenuation and the transmission distance affects the received signal intensity. Generally, the received signal intensity is inversely correlated with the transmission distance [10]. If the minimum power of receiving a packet is $P_{0}$, when the condition $P_{r}\geq P_{0}$ with an acceptable probability, Prob=90%, two sensor nodes can communicate normally [29]. Therefore, the probability $Prob\left(P_{r}\geq P_{0}\right)$ is given by:

(7) $$Prob\left(P_{r}\geq P_{0}\right)=Prob\left(\ell P_{t}C{d_{ij}}^{-2}\geq P_{0}\right)=Prob\left(\ell \geq \frac{P_{0}{d_{ij}}^{2}}{P_{t}C}\right)=\int_{\frac{P_{0}{d_{ij}}^{2}}{P_{t}C}}^{\infty }e^{-x}d_{x}=e^{-\frac{P_{0}{d_{ij}}^{2}}{P_{t}C}}$$

The maximum effective communication distance $d_{0}$ can be calculated as follow:

(8) $$Prob\left(P_{r}\geq P_{0}\right)=e^{-\frac{P_{0}{d_{0}}^{2}}{P_{t}C}}=0.9$$

(9) $$d_{0}=\sqrt{-\frac{CP_{t}ln0.9}{P_{0}}}$$

**Definition** **1.** *The neighbor nodes set of node *i* is:*

(10) $$NE\left(i\right)=\left\{j\left|d_{ij}\leq d_{0}\right.\right\}$$

#### 3.2.2. Forward Transmission Nodes

**Figure 5:**
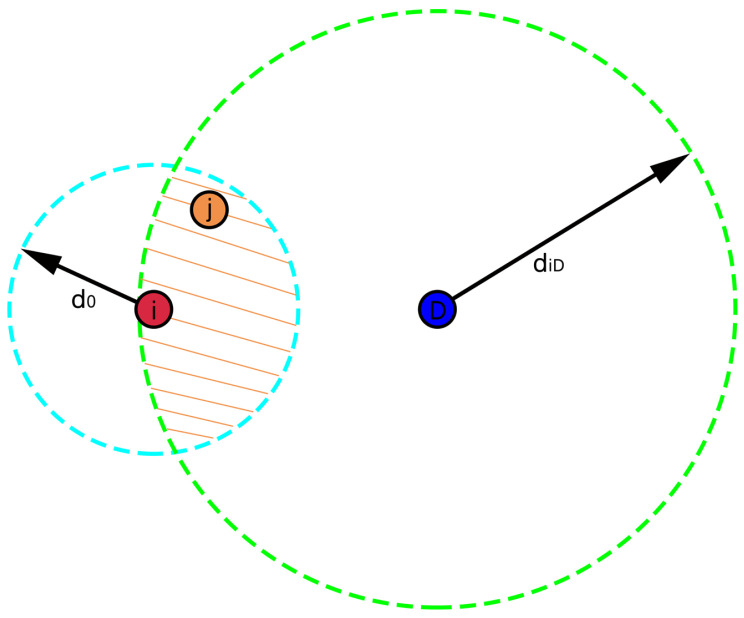
Forward transmission region.

To avoid routing loops, the selected node should be closer to the destination than the previous one. Therefore, it is necessary to control the direction of each hop forwarding to the destination node. For these, we define a forward transmission region as follows:

As shown in Figure 5, the two circles are made with node *i* as the center, $d_{0}$ as the radius, and the node *D* (destination node) as the center, $d_{iD}$ (the distance from node *i* to node *D*) as the radius, respectively. The area where the two circles intersect is the forward transmission region. Assuming the coordinate of *D* is (x,y). If node *j* is a forward transmission node of *i*, it should satisfy two conditions: firstly, *j* is within the transmission range of *i*; secondly, node *j* is closer to the destination *D* than *i*, that is:

(11) $$\left\{\begin{array}{c} {\left(x_{j}-x_{i})\right.}^{2}+{\left(y_{j}-y_{i})\right.}^{2}\leq {d_{0}}^{2}\\ {\left(x_{j}-x)\right.}^{2}+{\left(y_{j}-y))\right.}^{2}\leq {d_{iD}}^{2} \end{array}\right.$$

**Definition** **2.** *The forward transmission nodes set of node *i* is:*

(12) $$FN\left(i\right)=\left\{j\left|{\left(x_{j}-x_{i})\right.}^{2}+{\left(y_{j}-y_{i})\right.}^{2}\leq {d_{0}}^{2},{\left(x_{j}-x)\right.}^{2}+{\left(y_{j}-y)\right.}^{2}\leq {d_{iD}}^{2}\right.\right\}$$

#### 3.2.3. Candidate Nodes

If node *j* is a candidate node of node *i*, node *j* must meet the following conditions:

- Node *j* is a neighbor node of *i*. That is, the distance from *j* to *i* is less than the maximum transmission distance.
- Node *j* is a forward transmission node of node *i*.
- Node *j* can receive packets, and it is not a void node, i.e., $Flag_{j}=1$,$VFlag_{j}=0$.

In summary, the conditions that candidate nodes must fulfill:

(13) $$\left\{\begin{array}{c} d_{ij}=\sqrt{{\left(x_{j}-x_{i})\right.}^{2}+{\left(y_{j}-y_{i})\right.}^{2}}<d_{0}\\ {\left(x_{j}-x)\right.}^{2}+{\left(y_{j}-y)\right.}^{2}<{d_{\mathrm{iD}}}^{2}\\ Flag_{j}=1,VFlag_{j}=0 \end{array}\right.$$

**Definition** **3.** *The candidate nodes set for node *i* is:*

(14) $$CN\left(i\right)=\left\{j\left|j\in FN\left(i\right),Flag_{j}=1,VFlag_{j}=0\right.\right\}$$

### 3.3. Reception State Adjustment Mechanism

**Figure 6:**
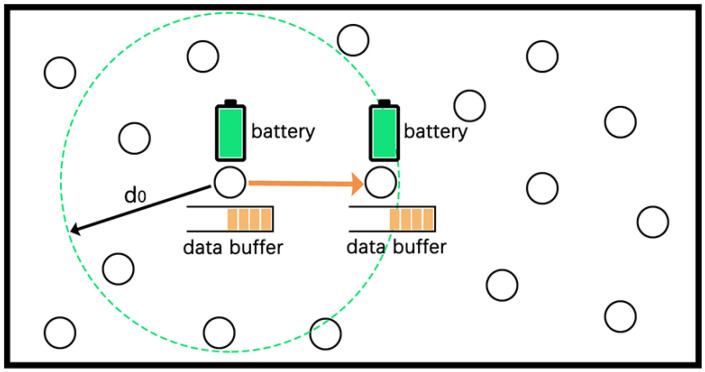
Reception state adjustment mechanism.

As shown in Figure 6, each node has a data buffer and a battery. Packets in the data buffer come from two sources, including newly generated packets by themselves (denoted as $C_{i}$) and packets received from other nodes (denoted as $I_{i}$). For the packets generated by nodes following Poisson process, $C_{i}$ obeying an exponential distribution and the value can be obtained by efficient prediction [30].

The number of newly generated packets by node *i* at time slot *t* can be written as:

(15) $$IC_{i}^{t}=I_{i}^{t}+C_{i}^{t}$$

The total amounts of packets in node *i* at the current time slot (denoted as *t*) can be calculated as:

(16) $$B_{i}^{t}=min\left\{B_{i}^{t-1}+IC_{i}^{t-1}-O_{i}^{t-1},DB_{max}/L\right\}$$

where $O_{i}^{t-1}$ denotes the number of packets processed by node *i* at time slot t−1.

When the data processing capacity of node reaches the upper bound (receiving too many packets), these packets have to stay in the buffer. In this case, a congestion problem would be caused easily, which accordingly increases the packet loss rate, reduces data processing efficiency and wastes energy [16,31]. In addition, although the energy harvesting device can provide unlimited energy, the nodes would suffer from occasional energy shortage as the harvested and demanded energy do not meet. When the node runs out of energy (i.e., entering the sleep mode), these remaining packets have to wait until the energy recovers to a certain threshold. Under this certain situation, it would lead to increasing packet transmission time and diminishing of the packet processing efficiency. In order to solve the above problems, we propose a reception state adjustment mechanism, as shown in Algorithm 1.

This adjustment mechanism uses Flag to indicate whether the node can receive packets. Flag=1 denotes node can receive data, and Flag=0 denotes cannot. There are three main parts:

(1) When the residual energy of a node is less than the minimum limit (Level1), the Flag would be adjusted to 0 if it is 1 (representing this node cannot receive packets). When the energy recovers to a certain threshold (Level2), the Flag would be adjusted to 1 if it is 0 (representing this node can continue to receive packets), that is:
  (17) $$\left\{\begin{array}{c} E_{r}^{i}\geq Level\;2,Flag=1\\ E_{r}^{i}\leq Level\;1,Flag=0 \end{array}\right.$$
(2) When the data buffer of a node reaches the maximum limit $DB_{th1}$, Flag would be adjusted to 0 if it is 1. When the buffer capacity is less than the lowest threshold $DB_{th2}$, Flag would be adjusted to 1 if it is 0, that is:
  (18) $$\left\{\begin{array}{c} DB\geq DB_{th1},Flag=0\\ DB\leq DB_{th2},Flag=1 \end{array}\right.$$
(3) When the residual energy of a node is not enough to forward the packets in the buffer, Flag would be adjusted to 0 if it is 1.

In this case, it needs to predict the total energy that the node can collect in a future period, as well as the energy required to process the packets in the buffer, so as to determine whether the node can process all packets in the buffer before its energy is exhausted.

(19) $$\left\{\begin{array}{c} B*E_{t}\geq E_{r}+EH_{i}*T+c_{1},Flag=0\\ B*E_{t}\leq E_{r}+EH_{i}*T-c_{2},Flag=1 \end{array}\right.$$

where *B* represents the number of packets in the buffer, B=DB/L, $c_{1}$ and $c_{2}$ are two constants. *T* represents the time required for the node to process all the packets in its buffer.

Since a packet must wait for the transmission of previous packets to complete, for a newly arrived packet in node *j*, we calculate its waiting time as:

(20) $$T_{j}=\sum_{num=1}^{N}t_{num}$$

where *N* denotes the number of packets in the current node. $t_{num}$ is the time required to transmit a packet when it has none of the previous packets, and it can be further expressed as:

(21) $$t_{num}=\sum_{k\in CN(j)}t_{\mathrm{jk}}*P_{jk}$$

*k* is a candidate node of *j*. $t_{jk}$ represents the time required for node *j* to forward a packet to node *k*. CN(j) denotes the set of candidate nodes of *j*. $P_{jk}$ indicates the probability that node *j* selects node *k* as the next hop.

**Figure 7:**
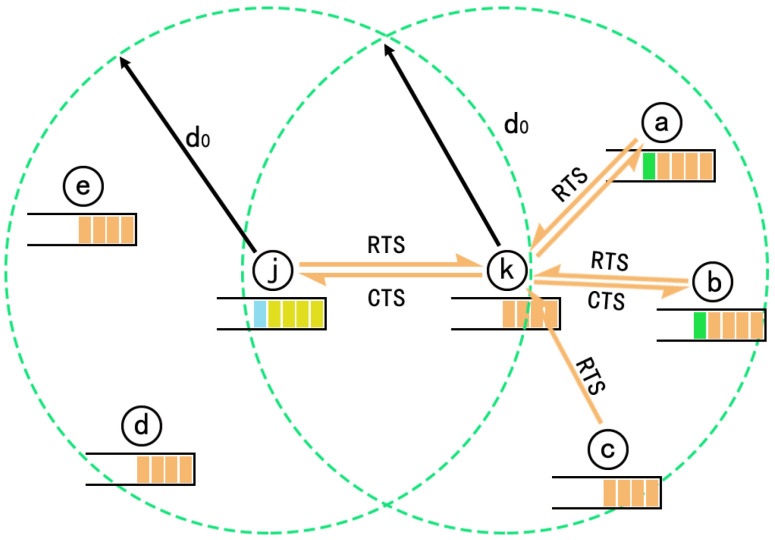
Interfering nodes.

According to the IEEE 802.11b protocol [32], when a node prepares to transmit a packet, it must ensure there is no other interfering node sending simultaneously [33,34,35]. At the same time, it cannot interfere with the nearby receiver [17]. It should be noted that interfering nodes include exposed nodes and hidden nodes [36] as shown in Figure 7.

If there is no other interfering node, the time required to transmit a packet from a node to its neighbor immediately is $t_{unit}=L⁄R+t_{0}$, where $t_{0}$ is the time required to exchange RTS, CTS and ACK frames [37].

Based on the above analysis, if node *j* prepares to send a data packet to node *k*, it must wait until $z_{jk}$ packets of other interfering nodes have been completed. The time taken by node *i* sending a packet to node *k* is the sum of two terms: (i) the time required waiting for other interference nodes to send $z_{jk}$ packets $\left(z_{jk}*t_{unit}\right)$, (ii) the time required for the current node to send a packet $\left(t_{unit}\right)$. Thus:

(22) $$t_{jk}=\left(z_{jk}+1\right)*t_{unit}$$

Let the probability that node j sends a packet immediately after the communication channel becomes idle be $prob_{j\_k}$, then we can obtain:

(23) $$P\left(z_{jk}=n\right)=\left(1-prob_{j\_k}{)}^{n}prob_{j\_k}\right.$$

(24) $$E\left(z_{jk}\right)=\frac{1-prob_{j\_k}}{prob_{j\_k}}$$

(25) $$E\left(t_{jk}\right)=\left[E\left(z_{jk}\right)+1\right]t_{unit}=\frac{t_{unit}}{prob_{j\_k}}$$

According to [30], assuming $\alpha_{jk}$ and $\beta_{jk}$ are the idle probabilities of the node *j*’s and node *k*’s neighbor nodes, respectively, then $prob_{j\_k}$ can be represented as:

(26) $$prob_{j\_k}=\alpha_{jk}*\beta_{jk}=\prod_{a\in \varepsilon_{j}}\left(1-\varphi_{a}\right)*\prod_{b\in \varepsilon_{k}}\left(1-\psi_{b}\right)$$

where $\varphi_{a}$ is the proportion of time taken by node *a* receiving packet; $\psi_{b}$ denotes the proportion of time taken by node *b* transmitting packets; $\varepsilon_{j}$ and $\varepsilon_{k}$ represent the interference node sets of node *j* and *k*, respectively.
**Algorithm 1:**Reception State Adjustment Mechanism.**Require:** The current node *i*; Residual energy $E_{r}^{i}$; Utilized cache size DB; The candidate nodes set of i CN(i); Current data receive state of i Flag1: **if** $E_{r}^{i}\geq Level\;2$ && $DB\leq DB_{th2}$ && $DB/L*E_{t}\leq E_{r}+EH_{i}*T-B$ **then**2:   **if** Flag=0 **then**3:      Flag=14:   **end if**5: **else**6:   **if** $E_{r}^{i}\leq Level\;1∥DB\geq DB_{th1}∥DB/L*E_{t}\geq E_{r}+EH_{i}*T+A$ **then**7:     **if** Flag=1 **then**8:         Flag=09:     **end if**10:   **end if**11:**end if**


## 4. GS-EERA

In this section, we provide the detailed process of the proposed algorithm, GS-EERA. Furthermore, we prove the correctness and computational complexity of this algorithm.

### 4.1. The Next Hop Selection

To obtain a suitable relay node, we fully consider the residual energy, energy harvesting rate, distance and data transmission time to define a metric and adopt the greedy strategy to select the optimum next hop from the candidate node(s). The metric is defined as follow:

(27) $$M_{ij}=\alpha {\hat{E}}_{r}^{j}+\beta {\hat{EH}}_{j}+\gamma \left(1-{\hat{D}}_{j}\right)+\delta \left(1-{\hat{T}}_{j}\right)$$

where α, β, γ and δ are the weighting coefficients. $E_{r}$, EH, *D* and *T* denote the residual energy, energy harvesting rate, distance and transmission time, respectively. $\hat{E}$, $\hat{EH}$, $\hat{D}$ and $\hat{T}$ represent the normalized functions, which meet the following rules:

(28) $$\left\{\begin{array}{c} \alpha +\beta +\gamma +\delta =1\\ {\hat{E}}_{r}^{j}=\frac{E_{r}^{j}}{E_{m}};\;\;\;{\hat{EH}}_{j}=\frac{EH_{j}}{EH_{max}}\\ {\hat{D}}_{j}=\frac{D_{jd}}{D_{id}};\;\;\;{\hat{T}}_{j}=\frac{T_{j}}{\sum_{a\in CN(i)}T_{a}} \end{array}\right.$$

where $E_{m}$ and $EH_{max}$ indicate the maximum value of residual energy and energy harvesting rate, respectively.

Therefore, the probability that node *i* chooses node *j* as the next hop can be calculated as:

(29) $$P_{\mathrm{ij}}=\frac{M_{ij}}{\sum_{j\in CN(i)}M_{ij}}$$

**Figure 8:**
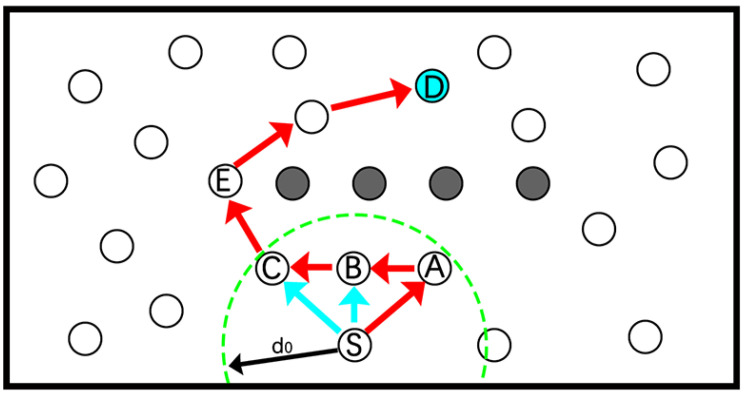
Routing hole problem.

The packets may encounter a void node during transmission process. In WSN, the increasing number of nodes would extend data transmission time, waste energy and even cause transmission failure. When encountering a routing hole, the classical GPSR algorithm [19] takes the perimeter forwarding strategy, traverses the closest planar sub-image and forwards the data along the boundary of the void area using the right-hand rule. As far as we know, it is not the optimal selection in some cases. Let us take an example, as shown in Figure 8, *S* is the source node, and *A*, *B* and *C* are the neighbor nodes of *S*. Firstly, the data are forwarded to node *A* through the greedy forwarding method. Since node *A* is a void node, it adopts the perimeter forwarding strategy to relay the data bypass the routing void area along A→B→C→D. Obviously, if node *S* forwards data along S→B→C→D or S→C→D, the total energy overhead and the data transmission time cost may be relatively less.

In this paper, we propose a method that combines void recovery and void avoidance to deal with the routing hole problem. If the candidate nodes set of a node is empty, the VFlag is adjusted to 1 (indicating this is a void node and cannot become a candidate node of others), the RFlag of the packet to be processed at this time is adjusted to 1 (i.e., the node should forward packet through the perimeter forwarding strategy) and selects the optimal next hop from its neighbor nodes with Flag=1. If a non-void node receives a packet with RFlag=1, then RFlag is adjusted to 0 (i.e., stopping the perimeter forwarding process and starting the greedy strategy-based packets forwarding).

### 4.2. Algorithm Procedure

Step 1 (initialization): Each node broadcasts an initial packet with its location information, and then calculates the distance to the other nodes.

Step 2 (updating node information): At the beginning of each time slot, each node exchanges the state information with its neighbor nodes and updates the information in the Neighbor Table.

Step 3 (determining the next hop): When the destination is within the communication range of current node, the destination would be selected as the next hop directly;

When the current node is not a void node, and the RFlag of the packet to be processed at this time is 1, the RFlag would be adjusted to 0, and the optimal next hop would be selected from the candidate nodes set according to Formula (27);

When the current node is a void node, the VFlag would be adjusted to 0 if it is 1 (i.e., this node cannot become a candidate node of others) and adjusts the RFlag of the packet to be processed at this time to 1. Then, takes the perimeter forwarding strategy to select the optimal next hop from its neighbor nodes which Flag=1.

Step 4: Packet is sent to the next hop.

Step 5: Steps 2, 3 and 4 are repeated until the packet is received by destination node.

**Figure 9:**
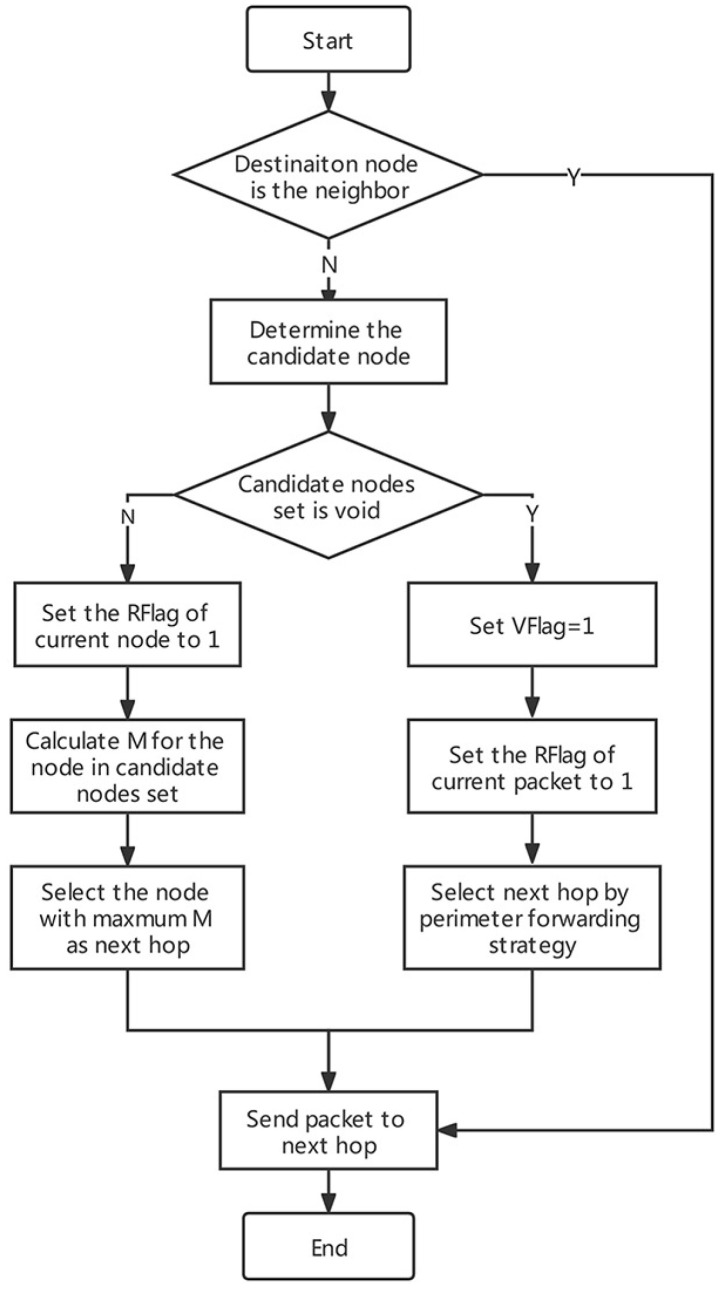
The procedure of GS-EERA main algorithm.

The procedure of main algorithms of GS-EERA is presented in Figure 9, and the pseudocode of GS-EERA is shown in Algorithm  2.
**Algorithm 2:**GS-EERA.**Require:** The current node *i*; The destination node *D*; The neighbor nodes set of i NE(i); CN=ϕ1: **for all**
j∈NE(i)
**do**2:   Execute Alg.13:   **if**
Flag=1
**then**4:        CN(i)=CN(i)∪j5:   **end if**6: **end for**7: **if** 
D∈NE(i)
**then**8:   Relay=D9: **else**10:   **if** CN(i)∉ϕ **then**11:        Set the RFlag of current packet to 0.12:        **for all**
j∈CN(i)
**do**13:         Calculate $M_{ij}$            find the node with maximum $M_{ij}.$            
Relay=j14:      **end for**15:   **else**16:      VFlag=1            Set the RFlag of current packet to 1.            Select next hop from neighbor nodes with Flag=1 by perimeter forwarding strategy17:   **end if**18: **end if**
     RETURN Relay

### 4.3. Algorithm Analysis

#### 4.3.1. Correctness Analysis

According to our algorithm, GS-EERA, it selects next hop with the maximum *M* from the candidate nodes set (Formula (27)). Let the average weight of the nodes in a path be ${\overset{-}{M}}_{S,D}=\sqrt[{m}]{\prod_{i=1}^{m}M_{i}}$, where *m* is the number of hops from source node *S* to destination node *D*. A routing selection strategy is the optimal if it can select the route with the largest ${\overset{-}{M}}_{S,D}$ among all routes from *S* to *D*.

If the first k−1 hops of the selected nodes are already the optimal route, i.e., ${\overset{-}{M}}_{S,P}=\sqrt[{k-1}]{\prod_{i=1}^{k-1}M_{i}}$ (where *P* is the k−1st node) is the maximum among all routes from *S* to *P*. Assume that *R* is the node with maximum *M* in the candidate nodes set of *P*. In the kth hop route selection, if using GS-EERA, *P* selects *R* as the next hop and ${\overset{-}{M}}_{S,R}=\sqrt[{k}]{\prod_{i=1}^{k}M_{i}}$. Suppose another superior strategy exists which selects $R^{\prime }$ as relay node (i.e., ${\overset{-}{M}}_{S,R^{{}^{\prime }}}$ is larger than ${\overset{-}{M}}_{S,R}$),

(30) $${\overset{-}{M}}_{S,R^{{}^{\prime }}}>{\overset{-}{M}}_{S,R}\underset{}{\Rightarrow }\sqrt[{k}]{\prod_{i=1}^{k}{M^{{}^{\prime }}}_{i}}>\sqrt[{k}]{\prod_{i=1}^{k}M_{i}}\underset{}{\Rightarrow }{M^{{}^{\prime }}}_{k}>M_{k}$$

that is, the *M* of node $R^{\prime }$ is greater than *R*. It contradicts our earlier assertion that *R* is the node with maximum *M* among the candidate nodes set of *P*. Hence, there is no better strategy than GS-EERA. In fact, we can also see from the above derivation that if a superior strategy exists, that strategy selects the node with the largest *M* at every next-hop selection, which is exactly the same as the solution of GS-EERA. As a consequence, we conclude that GS-EERA is the optimal strategy.

#### 4.3.2. Complexity Analysis

The given approach, GS-EERA, has different time complexity. Firstly, at the initial time, each node calculates the Euclidean distance to the other nodes separately, which takes time as $O\left(n^{2}\right)$. Another key parameter of GS-EERA is the number of iterations. Suppose a single node in the network has at most *m* neighbor nodes. In the worst case, the network is linear. The process will be repeated for n−1 times and the time taken $O\left(mn\right)$. Therefore, the total time complexity of GS-EERA can write $O\left(n^{2}\right)+O\left(mn\right)=O\left(n^{2}\right)$.

## 5. Simulation Results and Analysis

In this section, we use the MATLAB platform to depict the performance of our algorithm, GS-EERA, under different parameter settings and compare them with SAGREH [8], EHR [6] and EHPRP [11].

### 5.1. Simulation Parameters Setting

The simulation experiments are conducted on MATLAB 2018a, and the PC configurations are Intel(R) Core(TM) i5-1135G7 2.40GHz, 8GB RAM, Windows 10 operating system. The specific network parameter settings are shown in Table 2. In this simulation, sensor nodes are randomly deployed in a square field of 200 × 200 m^2^, each node with the same initial energy $\left(E_{0}\right)$ of 5~15 J. The battery capacity $E_{m}=20\;J$ and the two thresholds for battery energy (Level1 and Level2) are set to 5 and $15\%E_{m}$, respectively (empirical values [1]). The energy consumption is caused by three parts: (1) sending packets (including initial information packets, ACK packets, reply packets and the data packets), (2) receiving packets (corresponds to the energy of sending packets) and (3) dissipation energy consumption of hardware for maintaining the node operation (including the energy required for modulation, coding, listening, etc.).

The data buffer can contain at most 20~60 packets $\left(B_{max}\right)$. Buffer upper threshold $\left(B_{upper}\right)$ is the boundary value that adjusts reception state from the receive mode to no-receive mode. If $B_{upper}$ is too small, it would result in buffer space wastage. While too large, it would easily cause packet loss. Similarly, buffer lower threshold $\left(B_{lower}\right)$ is the boundary value that adjusts the reception state from the no-receive mode to receive mode. If $B_{lower}$ is too large, it may cause the node to adjust its data reception state frequently, resulting in energy wastage. While too small, the node may have to wait for a long time duration to recover to the receive mode. In general, $B_{upper}$ and $B_{lower}$ are set to 90 and $70\%DB_{max}$, respectively (empirical values).

Data packets are generated by nodes following Poisson process with average packets generation rate (PGR) of 1~5 packets/s. To ensure the fairness of measurement factors, we set the four weighting factors in Formula (27) to be equal.

### 5.2. Simulation Metrics

(1) Packet delivery ratio (PDR). It is measured using the ratio of the number of packets received by destination to the number of packets transmitted by source.

(31) $$PDR=\frac{number\;of\;packets\;received\;by\;destination}{number\;of\;packets\;sent\;by\;source}$$

(2) Network energy variance. The average network residual energy can be calculated as:

(32) $${\bar{E}}_{r}\left(t\right)=\sum_{i=1}^{n}E_{r}^{i}\left(t\right)$$

The residual energy variance of the network can be expressed as:

(33) $$D\left(t\right)=\frac{1}{n}{\sum_{i=1}^{n}\left(E_{r}^{i}\left(t\right)-{\bar{E}}_{r}\left(t\right)\right)}^{2}$$

(3) Average end-to-end delay. It is the average time that a packet sends from source to destination.

(34) $$T=\frac{1}{N_{s}}\sum_{i=1}^{N_{s}}\left(t_{r}^{i}-t_{s}^{i}\right)$$

(4) Average energy consumption. The ratio of total energy consumption by network to the number of packets successfully received by the destination.

(5) Average hop count. In multi-hop networks, the routing hop affects the transmission delay. Therefore, it also reflects network performance to some extent.

We measure the performance of routing algorithms from five aspects: (1) the impact of the number of nodes, (2) the impact of initial energy, (3) the impact of simulation time, (4) the impact of buffer capacity, (5) the impact of packets generation rate, (6) the performance change with time and (7) the network recovery time.

### 5.3. Simulation Result

#### 5.3.1. The Impact of the Number of Nodes

**Figure 10:**
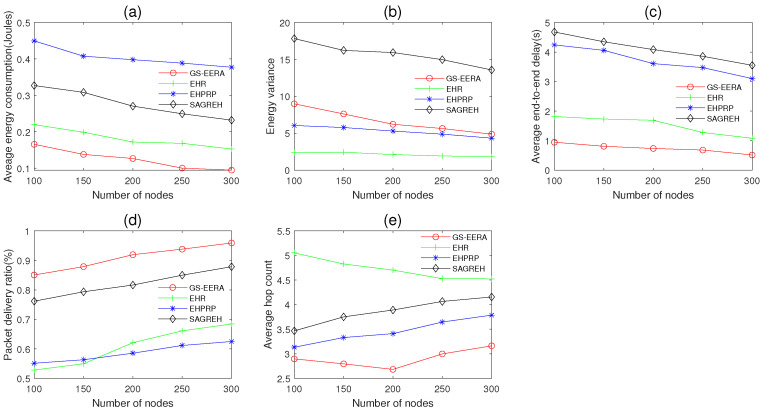
The impact of the number of nodes on (**a**) average energy consumption, (**b**) energy variance, (**c**) average end-to-end delay, (**d**) packet delivery ratio and (**e**) average hop count.

In this simulation group, we set the initial energy $E_{0}=15$ J, simulation time $T_{s}=600$ s, packets generation rate PGR=2 packets/s, data buffer capacity $B_{max}=40$ packets and the number of sensor nodes *n* varies in 100~300. Figure 10 shows the impact of *n* on average energy consumption, energy variance, average end-to-end delay, packet delivery ratio and average hop count.

Overall, as the number of nodes *n* increases, the energy consumption and end-to-end delay decrease (not absolutely). The reason is that when *n* increases, more optimal relay nodes with higher residual energy and lower transmission delay can be found (through executing the routing strategy), and the void nodes can be reduced. Thus, the increases in *n* can ease the congestion to some extent. Meanwhile, due to this reason, packet delivery ratio increases with the increasing *n*. For the energy variance, due to the negative relation between average energy consumption and *n* (nodes harvest the same amount of energy while consuming less), the energy difference between the nodes diminishes. Hence, energy variance is inversely correlated with the number of nodes. It should be explained that EHR always selects the relay node with the maximum energy state level. As a result, *n* does not have a significant impact on EHR’s energy variance. The number of the hop count is affected not only by *n* but also by the detailed routing strategy. Thus, the average hop count does not absolutely change with *n*.

From Figure 10a,c,d, we find that our algorithm, GS-EERA, achieves the optimum performance in average energy consumption, end-to-end delay and packet delivery ratio. Take the energy consumption as the example, GS-EERA outperforms the EHPRP, EHR and SAGREH by about 193.38, 52.86 and 98.93%, respectively. The reasons can be explained as follows: Firstly, our algorithm leverages the reception state adjustment mechanism to alleviate the congestion problem, which can reduce the energy wasting, transmission delay and packets loss. Secondly, it chooses relay node based on multi-factors, which considers the residual energy, energy harvesting rate, distance and transmission delay. Thirdly, it also adopts void recovery and void avoidance methods to deal with the routing hole problem. Therefore, it alleviates the issues of packets loss, long transmission delay and energy wasting brought by routing holes. EHR considers the buffer constraint problem. However, it neglects the impact of energy state, collision of wireless channel and the routing hole problem. Therefore, EHR only slightly eases congestion, and it performs worse than GS-EERA in terms of energy consumption and end-to-end delay. SAGREH determines the candidate route based on distance, direction and residual energy, i.e., choosing a transmission path with the highest reliability. Hence, SAGREH achieves acceptable performance in energy consumption and packet delivery ratio.

From Figure 10e, we see that the number of hop counts of GS-EERA is less than the other three routing algorithms. That is because GS-EERA makes use of the communication range judgement model to determine the candidate nodes, which ensures the optimal forward direction. Furthermore, it takes distance into account when selecting the relay node (Formula (27)), guaranteeing each hop as long as possible. SAGREH and EHPRP also comprehensively consider the direction and distance in the next hop selection. Nevertheless, these two algorithms do not control the direction and ignore the routing hole issue. Therefore, they have a relatively higher number of routing hops compared with GS-EERA. EHR performs poorly in average hop count as these two factors are not considered.

From Figure 10b, we observe that EHR achieves the best performance in energy variance, since it always selects relay nodes with the highest energy level and maximum energy harvesting density. GS-EERA and EHPRP consider muti-factors including the residual energy and energy harvesting rate. As a result, these two algorithms obtain relatively worse performance than EHR in energy variance. SAGREH does not efficiently consider the impact of energy state on next hop selection. Thus, it performs poorly in energy variance.

#### 5.3.2. The Impact of Initial Energy

**Figure 11:**
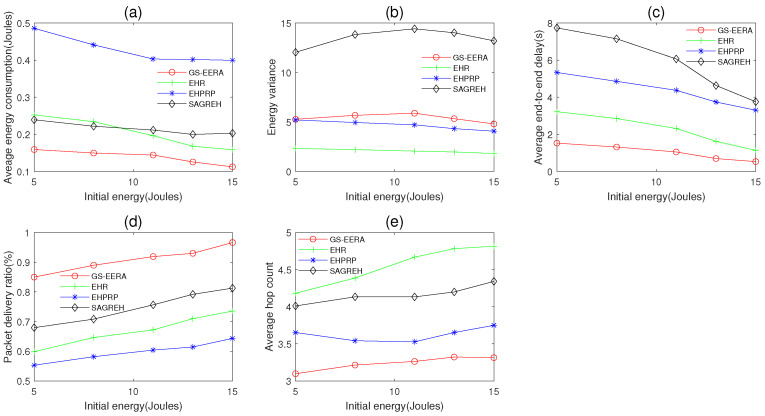
The impact of initial energy on (**a**) average energy consumption, (**b**) energy variance, (**c**) average end-to-end delay, (**d**) packet delivery ratio and (**e**) average hop count.

In this simulation group, we set the number of nodes n=300, simulation time $T_{s}=600$ s, packets generation rate PGR=2 packets/s, data buffer capacity $B_{max}=40\;\mathrm{packets}$ and the initial energy $E_{0}$ varies in 5~15 J. Figure 11 shows the impact of $E_{0}$ on average energy consumption, energy variance, average end-to-end delay, packet delivery ratio and average hop count.

Overall, as the initial energy $E_{0}$ increases, the energy consumption and end-to-end delay decrease, and the packets delivery rate increases. With more $E_{0}$, the nodes are easier to be in the active mode. Thus, the number of candidate nodes increases. As described in Section 5.3.1, the increasing of the number of candidate nodes increases can improve the performance of energy consumption, end-to-end delay and packet delivery ratio. The energy variance and average hop count are affected not only by the initial energy but also by the detailed routing strategy. As a result, these two metrics do not have clearly change rules.

GS-EERA still achieves the optimum performance in average energy consumption, end-to-end delay, packet delivery ratio and hop count, and acceptable performance in energy variance. Take the average hop count as an example, GS-EERA is 20.38% less than EHPRP, 32.12% less than SAGREH, and 45.73% less than EHR, respectively. Since EHR emphasizes the energy state of selected nodes and considers buffer constraints to alleviate congestion problem, it has good performance in energy consumption, energy variance and end-to-end delay. EHPRP achieves better performance in energy variance and average hop count, while performing worse in end-to-end delay and packet delivery ratio. SAGREH achieves acceptable performance in energy consumption, packet delivery ratio but performs badly in energy variance and end-to-end delay (the reasons have been explained in Section 5.3.1).

#### 5.3.3. The Impact of Simulation Time

**Figure 12:**
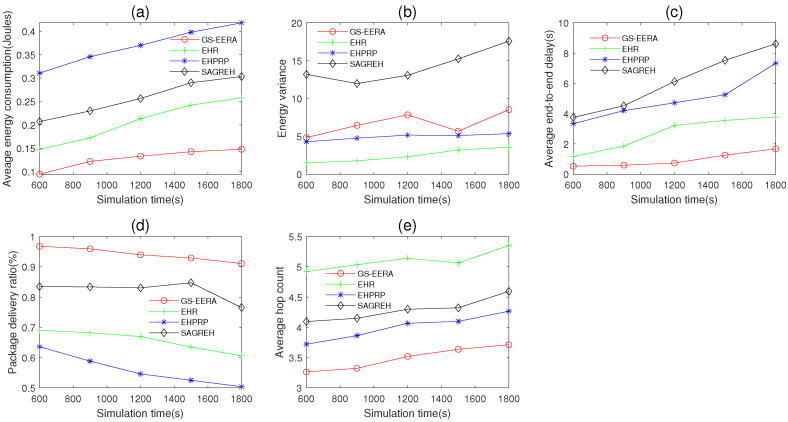
The impact of simulation time on (**a**) average energy consumption, (**b**) energy variance, (**c**) average end-to-end delay, (**d**) packet delivery ratio and (**e**) average hop count.

In this simulation group, we set the number of nodes n=300, initial energy $E_{0}=15$ J, packets generation rate PGR=2 packets/s, data buffer capacity $B_{max}=40$ packets and simulation time $T_{s}$ varies in 600~1800 s. Figure 12 shows the impact of $T_{s}$ on average energy consumption, energy variance, average end-to-end delay, packet delivery ratio and average hop count.

Overall, with the simulation time $T_{s}$ increases, energy consumption, end-to-end delay and hop count increase. The reason is that when $T_{s}$ increases, the residual energy and buffer size of more nodes reach up to the bound, and thereby they cannot forward packets. Accordingly, the data transmission is potentially interrupted. It should be noted that since EHR, EHPRP and SAGREH do not consider the relationship between energy state and data buffer capacity, the number of nodes which are in the sleep mode with unforwarded packets would increase with $T_{s}$. Therefore, the end-to-end delay of these three algorithms increases dramatically. In addition, for these reasons, the packet delivery ratio decreases with the increasing $T_{s}$. As $T_{s}$ increases, some nodes may have lower residual energy (at a fixed number of nodes, some nodes are repeatedly used for data transmission), while some nodes are used infrequently but still access the harvested energy. Hence, the energy variance increases.

Compared with the other three routing algorithms, GS-EERA still achieves the optimum performance in energy consumption, end-to-end delay, packet delivery ratio and hop count and acceptable performance in energy variance. Take the end-to-end delay as the example, GS-EERA is 334.21% less than EHPRP, 202.32% less than EHR and 365.27% less than SAGREH, respectively. EHR achieves good performance in energy consumption, energy variance and end-to-end delay but performs poorly in packet delivery ratio and average hop count. EHPRP achieves better performance in energy variance and average hop count, while obtaining worse energy consumption and packet delivery ratio. SAGREH achieves acceptable performance in the energy consumption and packet delivery ratio (the reasons have been explained in Section 5.3.1).

#### 5.3.4. The Impact of Data Buffer Capacity

**Figure 13:**
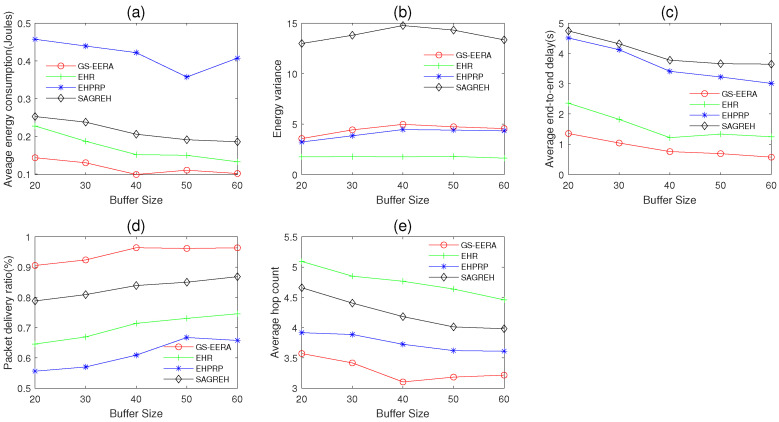
The impact of data buffer capacity on (**a**) average energy consumption, (**b**) energy variance, (**c**) average end-to-end delay, (**d**) packet delivery ratio and (**e**) average hop count.

In this simulation group, we set the number of nodes n=300, initial energy $E_{0}=15$ J, packets generation rate PGR=2 packets/s, simulation time $T_{s}=600$ s and the data buffer capacity $B_{max}$ varies in 20~60 packets. Figure 13 shows the impact of $B_{max}$ on average energy consumption, energy variance, average end-to-end delay, packet delivery ratio and average hop count.

Overall, as the data buffer capacity $B_{max}$ increases, the energy consumption, end-to-end delay and hop count decrease, while the packet delivery ratio increases. With the increase in $B_{max}$, the congestion issue can be effectively alleviated, more sensor nodes can receive data and the routing strategy may find an optimal relay node. As a consequence, the performance of energy consumption, end-to-end delay, packet delivery ratio and hop count are improved. The energy variance is affected by multiple factors. Thus, it does not have a clear change rule.

For GS-EERA, it still achieves the optimal performance in energy consumption, end-to-end delay, packet delivery ratio and hop count and acceptable performance in energy variance. The reason is that GS-EERA introduces a reception state adjustment mechanism, and thereby nodes can dynamically adjust packet reception according to the remaining buffer size and energy state (i.e., easing the congestion problem). Due to the mechanism, the performance of packet delivery ratio, energy consumption and end-to-end delay is also improved. Take the packet delivery ratio as an example, GS-EERA outperforms the EHPRP, EHR and SAGREH by about 34.41, 24.73 and 16.12%, respectively. EHR achieves good performance in energy consumption, energy variance and end-to-end delay. EHPRP achieves better performance in energy variance and average hop count, while worse in energy consumption and packet delivery ratio. SAGREH achieves acceptable performance in the energy consumption and packet delivery ratio but performs poorly in energy variance and end-to-end delay (the reasons have been explained in Section 5.3.1).

#### 5.3.5. The Impact of Packets Generation Rate

**Figure 14:**
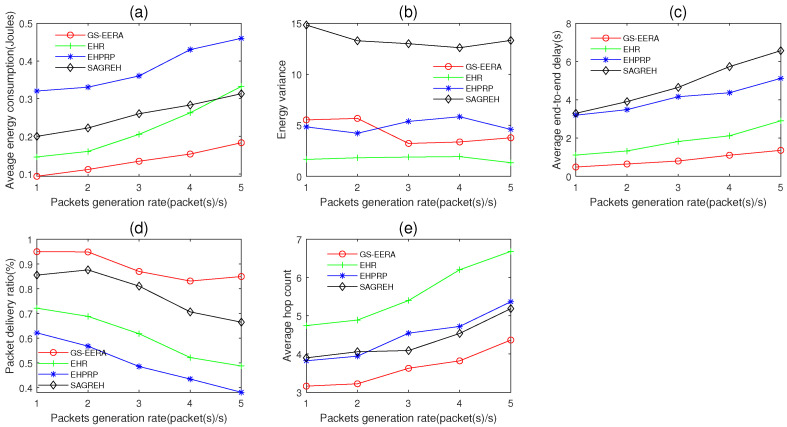
The impact of packets generation rate on (**a**) average energy consumption, (**b**) energy variance, (**c**) average end-to-end delay, (**d**) packet delivery ratio and (**e**) average hop count.

In this simulation group, we set the number of nodes n=300, initial energy $E_{0}=15$ J, simulation time $T_{s}=600$ s, data buffer capacity $B_{max}=40$ packets and the packets generation rate PGR varies in 1~5 packets/s. Figure 14 shows the impact of PGR on average energy consumption, energy variance, average end-to-end delay, packet delivery ratio and average hop count.

Overall, as the packets generation rate PGR increases, energy consumption, end-to-end delay and hop count increase and packet delivery ratio decreases (not absolutely). When PGR increases, there are more packets in the buffer of nodes, which intensify the packet transmission frequency. Congestion probability would increase if the frequency is too high, which decreases the network performance. In contrast, if PGR is lower, the generated packets can be processed in time and congestion probability is decreased. The energy variance is affected by multiple factors. Thus, it has an unclear change rule.

For GS-EERA, it still achieves the optimal performance in energy consumption, end-to-end delay, packet delivery ratio and hop count and acceptable performance in energy variance. That is because the introduced reception state adjustment mechanism can reduce the probability of congestion occurrence and enhance the efficiency of packet transmission. Take the average hop count as an example, GS-EERA is 22.85% less than EHPRP, 51.35% less than EHR and 17.18% less than SAGREH, respectively. EHR performs better in energy consumption, energy variance and end-to-end delay, while worse in packet delivery ratio and hop count. EHPRP achieves better performance in energy variance and average hop count, while underperforming in energy consumption and packet delivery ratio. SAGREH achieves acceptable performance in the energy consumption and packet delivery ratio but performs worse in energy variance and end-to-end delay (the reasons have been explained in Section 5.3.1).

#### 5.3.6. The Performance Change with Time

**Figure 15:**
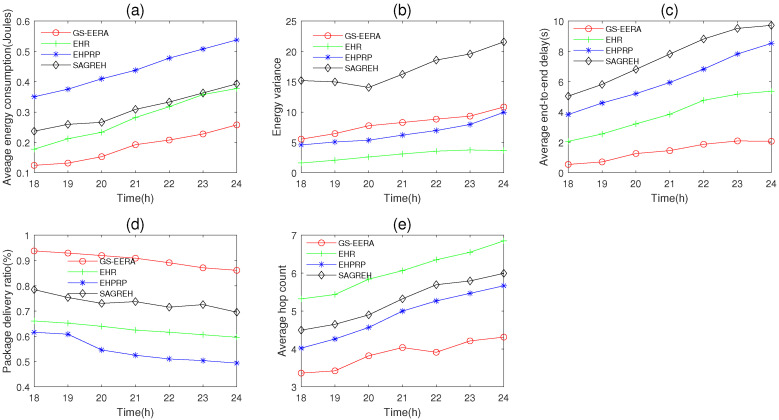
The performance change with time. (**a**) average energy consumption, (**b**) energy variance, (**c**) average end-to-end delay, (**d**) packet delivery ratio and (**e**) average hop count.

In this simulation group, we set the energy harvesting time from 18 to 24 h (Figure 3), in which energy input from the photovoltaic energy harvester falls down drastically, to observe the performance change of the routing algorithms. We set the number of nodes n=300, initial energy $E_{0}=15$ J, packets generation rate PGR=2 packets/s and data buffer capacity $B_{max}=40$ packets. Figure 15 shows the performance of average energy consumption, energy variance, average end-to-end delay, packet delivery ratio and average hop count change with time.

Overall, under conditions of the energy harvesting rate falling down drastically, with an increase in the simulation time, energy consumption, end-to-end delay, energy variance and hop count, the packet delivery ratio decreases. The reasons have been explained in Section 5.3.3. We can notice that the decrease in energy harvesting rate has relatively less impact on our algorithm (GS-EERA) than the other three algorithms. That is because the use of the reception state adjustment mechanism can ease the congestion issue and save energy. Therefore, our algorithm has good network stability.

Compared with the other three routing algorithms, GS-EERA still achieves the optimum performance in energy consumption, end-to-end delay, packet delivery ratio and hop count and acceptable performance in energy variance. Take the average hop count as an example, GS-EERA is 28.57% less than EHPRP, 42.12% less than SAGREH and 68.57% less than EHR. EHR achieves good performance in energy consumption, energy variance and end-to-end delay but performs poorly in packet delivery ratio and average hop count. EHPRP achieves better performance in energy variance and average hop count, while achieving a worse energy consumption and packet delivery ratio. SAGREH achieves acceptable performance in the energy consumption and packet delivery ratio (the reasons have been explained in Section 5.3.1).

#### 5.3.7. The Network Recovery Time

In this simulation group, we set 50 and 75% of the nodes’ initial energy as 0, and the others are 12 J. The photovoltaic energy harvesting technology is used, and the time set from 0 to 6 h (Figure 3). When 80% of nodes can receive packets, we view the network as recovery.

**Figure 16:**
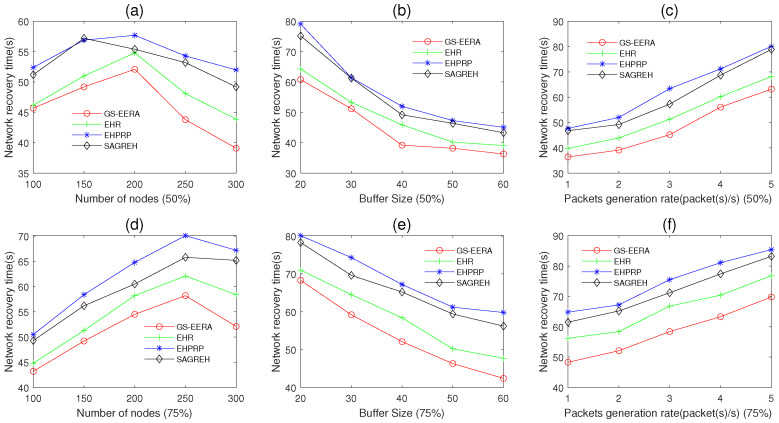
The network recovery time.

Figure 16a–c shows the performance of network recovery time when 50% of the nodes’ initial energy is 0. In Figure 16a, we set the data buffer capacity $B_{max}=40$ packets, packets generation rate PGR=2 packets/s and the number of nodes n varies in 100~300. Figure 16a shows the impact of *n* on network recovery time. Overall, with the increases in *n*, the network recovery time increases first, then decreases. The reason is that when *n* increases, the energy consumption of the network reduces (the reasons have been explained in Section 5.3.1) and there are fewer alive nodes entering sleep mode; hence, the network recovery time decreases. However, if *n* is too less, there are more isolated nodes and fewer packets are transmitted in the network, resulting in less energy consumption. Therefore, we can see from Figure 16a that the recovery time increases when *n* is less than 200. Our algorithm, GS-EERA, achieves the optimal performance in the network recovery time. That is because, compared with the other three algorithms, GS-EERA comprehensively considers the buffer constrain and routing hole problems. Through leveraging a reception state adjustment mechanism to ease the congestion problem and adopting the void recovery and void avoidance method to deal with the routing hole problem, it can reduce the energy consumption. Therefore, the network recovery time of GS-EERA is shorter than the others three algorithms.

In Figure 16b, we set the number of nodes n=300, packets generation rate PGR=2 packets/s and the data buffer capacity $B_{max}$ varies in 20~60 packets. Figure 16b shows the impact of $B_{max}$ on network recovery time. Overall, with the increases in $B_{max}$, the network recovery time decreases. When the $B_{max}$ is small, congestion is more likely to occur, resulting in more energy-wasting and longer network recovery time. As $B_{max}$ increases, the congestion problem can be alleviated, and energy consumption can be reduced. Hence, the network recovery time decreases.

In Figure 16c, we set the number of nodes n=300, the data buffer capacity $B_{max}=40$ packets and packets generation rate PGR varies in 1~5 packets/s. Figure 16c shows the impact of PGR on network recovery time. Overall, with the increases in PGR, the network recovery time increases. That is because the higher PGR would bring out a higher congestion probability, causing the network recovery time to be longer.

Figure 16d–f shows the performance of network recovery time when 75% of the nodes’ initial energy is 0. The changing trend is similar to Figure 16a–c, and GS-EERA still achieves the optimal performance. The reasons have been explained in Paragraph 2. However, overall, when the number of the node in which the initial energy is 0 adjusts from 50 to 75%, the network recovery time increases.

## 6. Conclusions

In order to improve energy efficiency and address the buffer constraint problem in EH-WSNs, we propose a brand new greedy strategy-based energy-efficient routing protocol, GS-EERA. In the system modeling process, we firstly formulate an energy evaluation model to determine the energy state of a node and construct a communication range judgment model to identify the effective transmission area. Next, we introduce a reception state adjustment mechanism. By considering the data buffer state and MAC layer protocol, this mechanism can dynamically adjust the data reception state of nodes. With this, we give the detailed execution process of GS-EERA. In addition, we analyze the correctness and computational complexity of this algorithm. Finally, we conduct the simulation experiment. Compared with EHR, EHPRP and SAGREH, our algorithm obtains the optimal performance in average energy consumption, average end-to-end delay, packet delivery ratio and average hop count and acceptable performance in energy variance. Our algorithm provides a meaningful solution for constructing green communication systems.

In future studies, we think the following research directions are valuable: (1) adapting our algorithms to vertically heterogeneous 3D environments, (2) considering the impact of Unmanned Aerial Vehicles on routing design and (3) extending our algorithm to multipath routing.

## Figures and Tables

**Table 1:** The list of core notations related to the system model.

Notation	Meaning
$E_{r}$	Residual energy
$E_{m}$	Battery maximum capacity
EH	Energy harvesting rate
$EH_{max}$	Maximum energy harvesting rate
$E_{0}$	Initial residual energy
$E_{tx}$	Energy consumption for sending a packet
$E_{rx}$	Energy consumption for receiving a packet
$E_{s}$	Dissipation energy consumption of hardware
DB	Data buffer occupancy
*B*	The number of packets in buffer
Flag	Buffer packet reception state mark
$P_{t}$	Transmission power
$P_{r}$	Receive power
$d_{0}$	Maximum communication distance
$d_{ij}$	Distance between node *i* and *j*
NE(i)	Neighbor nodes set of node *i*
FN(i)	Forward transmission nodes set of node *i*
CN(i)	Candidate nodes set of node *i*
*L*	Packet length
*R*	Data transmission rate

**Table 2:** Parameters setting.

Parameter	Value
Network size	200 × 200 m^2^
The number of nodes (n)	100~300
Simulation time $\left(T_{s}\right)$	600~1800 s
Battery capacity $\left(E_{m}\right)$	20 J
Level1	$5\%E_{m}$
Level2	$15\%E_{m}$
Initial energy $\left(E_{0}\right)$	5~15 J
Maximum energy harvesting rate $\left(EH_{max}\right)$	0.3 J/s
Data buffer capacity $\left(B_{max}\right)$	20~60 packets
Data buffer upper threshold $\left(B_{upper}\right)$	$90\%B_{max}$
Data buffer lower threshold $\left(B_{lower}\right)$	$70\%B_{max}$
Data packet size (L)	512 Bytes
Packets generation rate (PGR)	1~5 packets/s
Data transmission rate (R)	10 kb/s
Dissipation energy consumption of hardware $\left(E_{s}\right)$	0.1 J/s
Transmission power $\left(P_{t}\right)$	200 mW
Weighting coefficient	α=β=γ=δ=0.25
Time slot $\left(t_{slot}\right)$	0.1 s
